# Correlation between the sagittal spinopelvic alignment and degenerative lumbar spondylolisthesis: a retrospective study

**DOI:** 10.1186/s12891-018-2073-z

**Published:** 2018-05-16

**Authors:** Qi Lai, Tian Gao, Xin Lv, Xuqiang Liu, Zongmiao Wan, Min Dai, Bin Zhang, Tao Nie

**Affiliations:** Department of Orthopedics, The First Affiliated Hospital of Nanchang University, Artificial Joints Engineering and Technology Research Center of Jiangxi Province, Nanchang, Jiangxi 330006 People’s Republic of China

**Keywords:** Lumbar degenerative spondylolisthesis, Sagittal alignment, Lumbar lordosis, Pelvic incidence, L5

## Abstract

**Background:**

Pain and disability associated with degenerative lumbar spondylolisthesis (DLS) results in significant burden on both the patients’ quality of life and healthcare costs. Currently, there is controversy regarding the specificity of spinopelvic measures of sagittal plane alignment with respect to DLS. Moreover, the correlation among spinopelvic parameters of sagittal plane alignment remains to be clarified. Our aim in this study was to compare these measurements between patients with single-segment DLS at L5 and a control group with no history of DLS.

**Methods:**

Our study group was formed of 132 patients who underwent full length lateral view radiographs of the spine in a relaxed standing posture. Among these, DLS at L5 was identified in 72 patients, forming the DLS group, with no radiographic evidence of lumbar spine disease in the remaining 60 patients, forming the control group. The patient and control groups were balanced with regard to age and sex distribution. The following spinopelvic parameters of sagittal plane alignment were measured: angle of incidence (PI) and tilt (PT) of the pelvis; sacral slope (SS); thoracic kyphosis (TK); lumbar lordosis (LL); and the spinal sagittal vertical axis (SVA). The Meyerding grade of L5 slippage was quantified for each patient in the DLS group.

**Results:**

Measures of TK, PI, SS, and LL were significantly greater in the DLS than control group (*P* <  0.05), with no between-group difference in SVA and PT. In the DLS group, the grade of L5 slippage correlated with SS (*r* = 0.873, *P* <  0.0001), PI (*r* = 0.791, *P* <  0.0001) and LL (*r* = 0.790, P <  0.0001). Moreover, the measurement for SS correlated more strongly with the PI (*r* = 0.94, *P* <  0.01) than the LL (*r* = 0.69, *P* <  0.01).

**Conclusion:**

Measurements of SS, PI, and LL were specifically associated with DLS, with measurements correlating positively with the grade of slippage.

**Electronic supplementary material:**

The online version of this article (10.1186/s12891-018-2073-z) contains supplementary material, which is available to authorized users.

## Background

The definition of DLS has been gradually refined over time, from a simple slippage of the vertebral body, described in 1930, to a slippage due to the absence of the pedicle isthmus, known as a pseudo spondylolisthesis and described by MacNab [1] in 1950. In 1963, Newman [2] introduced the concept of degenerative spondylolisthesis, with an estimated 4.1% incidence rate of DLS [3]. The incidence of DLS among women is 400% greater than among men [4], with DLS being most prevalent among women over the age of 50 years, and usually affecting the L4-L5 segment, with a grade of slippage of I-III [5].

The pain and disability associated with degenerative lumbar spondylolisthesis (DLS) cause a significant burden both at the level of patients’ quality of life and healthcare costs. The aetiology of DLS is complex and remains controversial, although some researchers have attributed DLS to age, trauma, sustained weight-bearing, and congenital malformation [6–8]. Barrey et al. [9, 10] reported an increase in the angle of incidence (PI) and tilt (PT) of the pelvis among patients with DLS in comparison to healthy volunteers, combined with a decrease in the slope of the sacrum (SS) and lumbar lordosis (LL). In their review of the spinopelvic alignment parameters for 65 patients with DLS, Barrey et al. concluded that PI was a causative factor of DLS. In their comparison of spinopelvic alignment between 50 patients with DLS and a control group without DLS, Fuano et al. [11] also reported the PI to be greater among patients with DLS compared to a control group, with an associated increase in SS, the slope of L4 and L5, thoracic kyphosis (TK) and LL. Therefore, although both studies identified an increase in PI, the association between DLS and other parameters of spinopelvic alignment (LL, PT, and SS) is controversial. Therefore, our aim in this study was to clarify this issue through a comparison of the spinopelvic parameters of sagittal plane alignment among patients with a DLS at L5 and a control group with no radiographic evidence of DLS.

## Methods

### Study group

Our study group included both patients with a single-segment DLS at L5 and individuals without DLS who underwent radiographic examination of the spine over the same period. All prospective participants underwent a lateral view radiograph of the spine in a relaxed standing position, including C7 and the hips. The inclusion criteria for participants in the control group were an absence of spondylolysis and scoliosis. The inclusion criteria for the DLS group was confirmation of a single-segment L5 spondylolisthesis. The Meyerding grade [12] of the DLS was defined based on the percentage of slippage of the L5 vertebral body (grade 0, no slip; grade I, a 1–25% slip; grade II, a 25–50% slip; grade III, a 51–75% slip; and grade IV, a 75–100% slip). Individuals with a previous history of spinal or pelvic surgery, as well as those with a lumbosacral transitional vertebra, spinal deformity, tumour, infection, fractures, Any condition that could affect the sagittal plane alignment of the spine, such as abnormal BMI, were excluded.

Over the study period from January 2015 to December 2016, 72 patients (35 males and 37 females) with a L5 DLS and meeting our inclusion/exclusion criteria were identified through the Department of Orthopaedics at our hospital and enrolled in our study. The distribution of DLS grades among this group was as follows: grade I, 28 cases; grade II, 22 cases; grade III, 15 cases; and grade IV, 7 cases. The average age of our patient group was 55.11 ± 2.567 years (range, 43–66 years). Our control group included 60 individuals (28 males and 32 females), with an average age of 55.38 ± 2.02 years (range, 40–65 years). The age and sex distribution was comparable between the patient and control group.

### Measurement of the sagittal plane spinopelvic alignment parameters

**Fig. 1:**
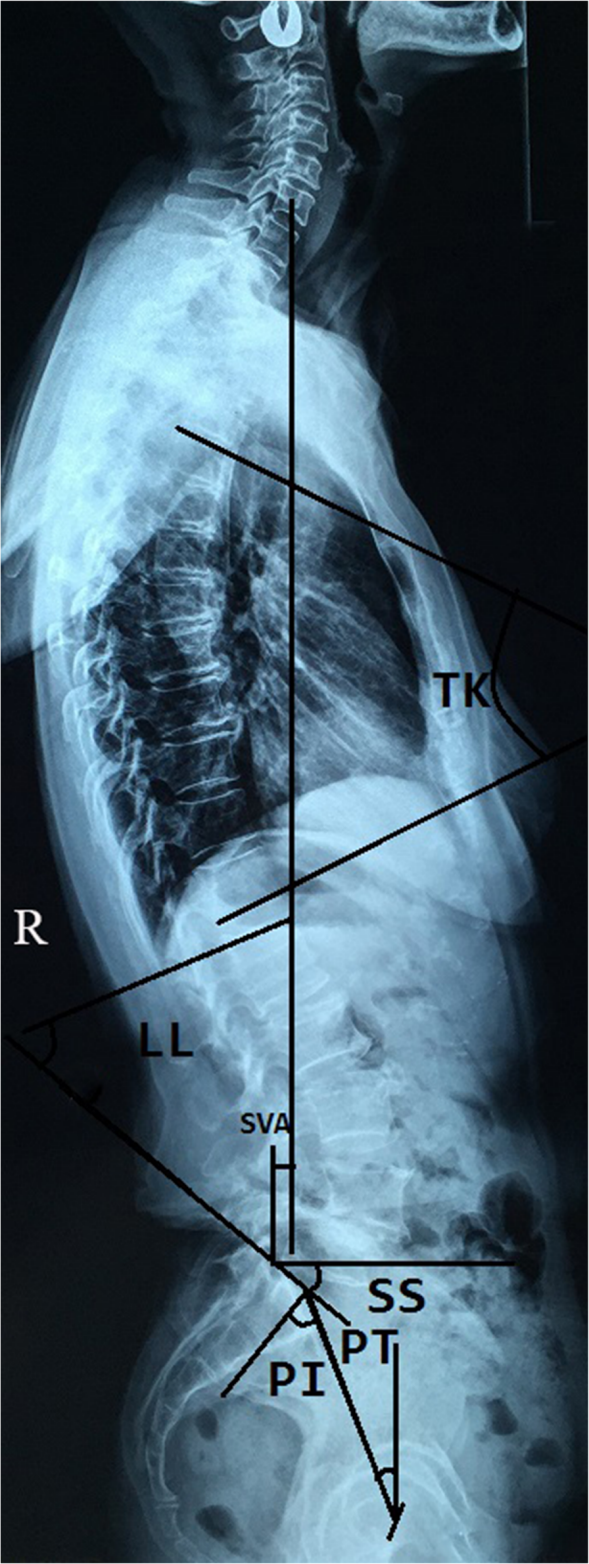
TK: The angle between the upper endplate of T4 and the lower endplate of T12; LL was measured using the Cobb angle between the superior end plate of the L1 and S1. PT was defined as the angle between a straight line connecting the midpoint of the bilateral femoral head centre to the midpoint of the sacral plate and the plumb line. PI was defined as the angle between the perpendicular line of the sacral plate and the line of the midpoint of the superior endplate of S1 joining with the centre of the hip axis. SS was defined as the angle formed by the upper endplate of S1 and the horizontal plane; SVA:C7 vertebral body along the vertical line and the upper edge of the S1 vertebral body

Spinopelvic parameters of sagittal plane alignment were measured from full-length lateral radiographs, obtained in a relaxed standing position (Fig. 1) The following parameters were measured: thoracic kyphosis (TK), defined as the angle between the upper endplate of T4 and the lower endplate of T12; lumbar lordosis (LL), defined as the Cobb angle between the superior endplate of L1 and S1; pelvic tilt angle (PT), defined as the angle between a straight line connecting the midpoint of the femoral head, bilaterally, and the midpoint of the sacral plate and the plumb line; pelvic incidence angle (PI), defined as the angle between a line perpendicular to the sacral plate and a line joining the midpoint of the superior endplate of S1 to the centre of the hip axis; sacral slope (SS), defined as the angle formed by the upper endplate of S1 and the horizontal plane; and the sagittal vertical axis (SVA), defined by the position of a plumb line, drawn from the vertebral body of C7, relative to the upper edge of the S1 vertebral body, with positive values indicative of the line passing through the anterior margin of S1 and negative values indicative of the line passing through the posterior margin of S1. All measurements were performed using our department’s Haitai Picture Archiving and Communication System (PACS). All parameters were measured by two physicians, to an accuracy of two decimals, with the average of the two measures used for analysis.

### Statistical analysis

All analyses were performed using Graphpad 5.0 statistical software. All data were expressed as the mean ± standard deviation, with the between-group difference for each alignment parameter evaluated using Student’s *t*-test. Correlations between two quantitative values were determined using the Pearson correlation coefficient. Additionally, One-way ANOVA could be performed to compare the value of PI, LL, SS, and TK in different DLS groups. If the value of these groups are different after the One-way ANOVA, Newman-Keuls test was used for post-hoc analysis. A *P*-value < 0.05 was considered statistically significant.

## Results

**Fig. 2:**
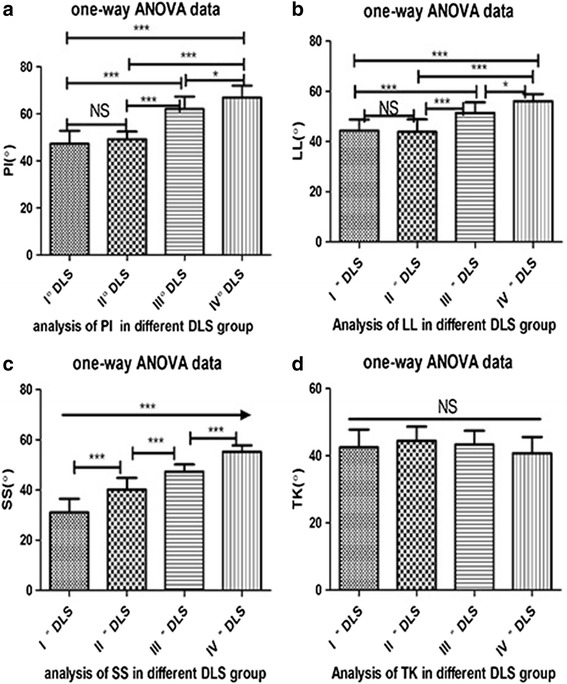
**a** represented that analysis result of PI in different DLS group via to one-way ANOVA; **b** represented that analysis result of LL in different DLS group via to one-way ANOVA; **c** represented that analysis result of ss in different DLS group via to one-way ANOVA; **d** represented that analysis result of TK in different DLS group via to one-way ANOVA; Additionally, "***" represented that *P* <0.0001;"**" represented that *P* <0.01;"*" represented that *P* <0.05;"NS" represented that *P* >0.05

Between-group differences in spinopelvic parameters are summarized in Table 1. Measures of PI, SS, TK, and LL were greater for the DLS than control group, with no between-group difference in PT and SVA. Then, the correlation between measured parameters and the DLS grade is reported in Table 2. PI, SS and LL correlated positively with the grade of L5 slippage as follows: SS (*r* = 0.873, *P* <  0.001); LL (*r* = 0.79, *P* <  0.001) and PI (*r* = 0.7916, *P* <  0.001). Additionally, via to One-way ANOVA analysis and Newman-Keuls as post-hoc test, the Fig. 2 displayed that the value of PI, SS and LL in grade I and II of DLS were obviously different compared to grade III or IV of DLS, with no between-group difference in TK. Finally, the correlation among spinopelvic parameters is summarised in Table 2. The degree of correlation among spinopelvic parameters differed between the DLS and control group, with SS being more strongly correlated with PI (*r* = 0.94, *P* <  0.01) than LL (*r* = 0.69, *P* <  0.01) in the DLS group, compared to stronger correlation with LL (*r* = 0.38, *P* <  0.01) than PI (*r* = 0.33, *P* <  0.01) in the control group.

**Table 1:** Comparison of the spinopelvic parameters between the 72 DS and 60 non-DS group (x ± s)

	DLS group	Normal group	*P* value
*n*	72	60	–
age	55.11 ± 2.567	55.38 ± 2.022	0.8165
SVA(mm)	24.87 ± 0.6580	25.44 ± 0.7107	0.5598
TK(°)	43.08 ± 0.5601	38.39 ± 0.9947	<0.0001
LL(°)	46.76 ± 0.7212	42.14 ± 0.7796	<0.0001
SS(°)	39.55 ± 1.090	35.47 ± 1.077	<0.0095
PT(°)	24.88 ± 0.5968	25.62 ± 0.6490	0.4023
PI(°)	52.85 ± 1.026	46.75 ± 0.8269	<0.0001

*DLS* degenerative lumbar spondylolisthesis, *DS* degenerative spondylolisthesis, *LL* lumbar lordosis, *PI* pelvic incidence, *PT* pelvic tilt, *SS* sacral slope, *SVA* sagittal vertical axis, *TK* thoracic kyphosis

**Table 2:** Correlation coefficients among the spinopelvic parameters in the DLS

	DLS group (*n* = 72)		Non-DLS group (*n* = 60)	
	*r* value	*P* value	*r* value	*P* value
TK - % Slip	−0.03507	0.7699	–	–
LL - % Slip	0.7900	< 0.0001	–	–
SS - % Slip	0.8730	< 0.0001	–	–
PI - % Slip	0.7916	< 0.0001	–	–
LL - PI	0.6546	4.457	0.1511	0.5988
LL - SS	0.6881	< 0.0001	0.3763	< 0.01
LL - TK	0.0688	0.565	0.1435	0.2290
PI - SS	0.9367	< 0.0001	0.3313	< 0.01
PI - TK	−0.0555	0.6432	0.0692	0.5988
TK - SS	0.03055	0.7988	−0.0290	0.8085

*DLS* degenerative lumbar spondylolisthesis, *LL* lumbar lordosis, *PI* pelvic incidence, *SS* sacral slope, *TK* thoracic kyphosis

## Discussion

Although the aetiology of DLS remains controversial, degenerative structural changes, including a decrease in intervertebral space, is commonly associated with a DLS. Specifically, these structural changes cause segmental instability which leads to a progressive slippage (or spondylolisthesis) of the affected vertebra. Progressive degenerative changes in the intervertebral disc, and associated changes in the structure and function of supporting muscles and ligaments of the spinal segment, may also play a role. More recently, changes in the line of force application on the spine, which cause changes in the sagittal plane alignment of the spine, have been implicated in the development and progression of DLS. In this regard, both anatomical measurements of alignment (PI) and postural measurements of alignment (LL, TK, PT, SS, and SVA) have been evaluated in relation to DLS [13–16]. Moreover, once a DLS develops, compensation in posture may lead to further change in spinopelvic parameters of sagittal plane balance and, consequently, progression of the DLS. Barrey et al. [9] proposed that an increase in the tilt angle of the L5 endplate, from normal reference values, was predictive of a spondylolisthesis, with an increase in PI being predictive of DLS. In agreement with these findings, Curylo et al. [17] reported a significantly greater PI (mean, 76.4°) among 53 patients with a lumbar spondylolisthesis compared to a control group. Labelle et al. [18] also reported greater values of LL and PT among patients with a lumbar spondylolisthesis, with an increase in these parameter values being positively correlated to the grade of spondylolisthesis. These studies confirm a relationship between DLS and spinopelvic parameters of sagittal plane alignment, with an increase in PI being specifically associated with DLS.

Despite previous studies, an increase in PI, and the association between DLS and other parameters of spinopelvic alignment (LL, PT, and SS) remains controversial [9–11]. Through our research, we similarly found that PI was associated with DLS. In addition, we also showed that among patients with DLS, TK, SS, and LL had higher values compared to a control group; however there were no between-group differences in SVA and PT. We also report a positive correlation between the grade of L5 slippage and PI, SS and LL (*P* <  0.01), with these parameter values increasing as a function of the grade of slippage. However, LL and PI values did not vary significantly among patients with a grade I or II DLS (Fig. 2a, b). It is possible that the degenerative changes in grade I and II DLS are mild and, therefore, not sufficient to influence sagittal plane alignment. Due to the relatively low number of patients in each of these two categories in our study group, verification of our findings in a larger population of patients with a grade I or II DLS is warranted. We also identified a difference in the correlation among the measured spinopelvic parameters for the DLS and control group. Specifically, in the DLS group, SS correlated more strongly with PI (*r* = 0.94) than LL (*r* = 0.69), while in the control group, SS correlated more closely to LL (*r* = 0.38) than PI (*r* = 0.33). Based on our findings, which are supported by a published report [11], we propose that PI is consistently associated with a DLS, with greater values of PI being associated with a higher grade of slippage. Measured changes in SS, LL and TK are likely to evolve as a function of the primary slippage of a vertebra.

**Fig. 3:**
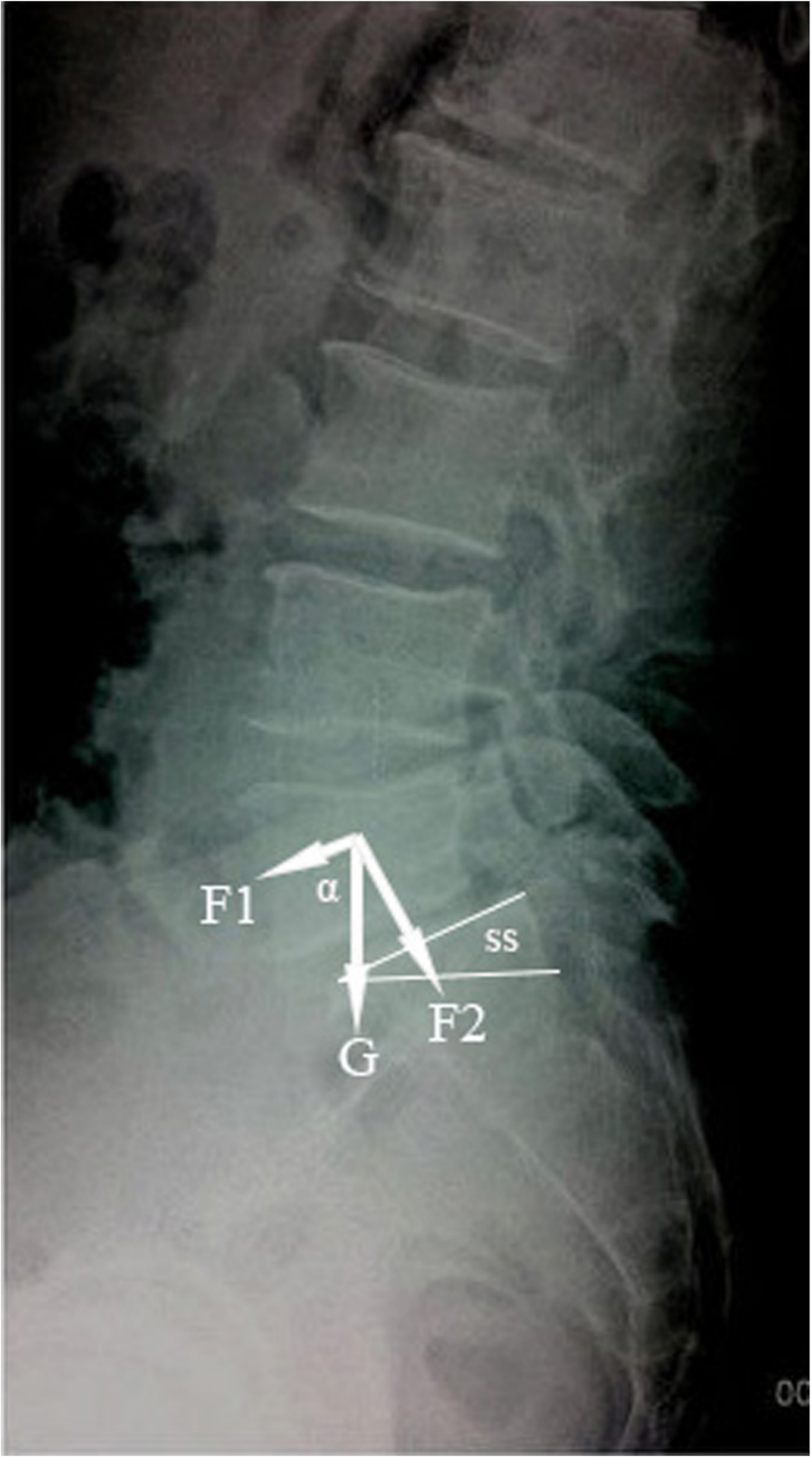
Vertebral mechanics analysis. G, gravity; F1, forward shear force, parallel to the end plate; F2, compression force exerted perpendicular to the end plate

Static equations of force on the lumbar vertebrae demonstrate the association between PI and SS in the DLS group. While standing, the force exerted on L5 can be composed into two force vectors, F1 being a shear force, parallel to the end plate of the vertebra, F2 being a compression force exerted perpendicular to the end plate (Fig. 3). Considering F1 to be equal to G*cos α, where α is angle between F1 and G, then F1 will be positively correlated to SS. As such, as SS increases, the magnitude of the forward shear force (F1) will also increase. As SS also correlates positively with PI (*r* = 0.94), PI, which is an invariable morphologic parameter of lumbosacral sagittal plane alignment, can be used to predict DLS [19], where PI = SS + PT [20]. Therefore, an increase in PI is a risk factor for DLS. The anterior slippage of L5 will explicitly cause a posterior displacement of the line of gravity of the lumbosacral spine and, therefore, an increase in LL. Although the effect of LL on DLS has been debated [21], we propose that the increase in LL is a secondary consequence of an anterior slippage of L5, rather than being a causative factor of DLS.

The limitations of our study need to be acknowledged in the interpretation of our results. Foremost, this is a retrospective study, with implicit selection bias. Moreover, although we identified a positive correlation between an increase in PI and DSL, we did not evaluate if DLS was more prevalent in a group of individuals with a higher PI. Therefore, the specific cause cannot be attributed to a higher PI, which would require a longitudinal study design. As well, we only included single-segment DLS involving L5, with no comparison to L4 spondylolisthesis, which might differentially influence measured spinopelvic parameters.

## Conclusions

Measurements of SS, PI, TK, and LL were specific to DLS, and were all significantly greater in the DLS group than in the control group. Moreover, measures of SS, PI, and LL positively correlated with the grade of L5 slippage in the DLS group. Among patients with a DLS, SS correlated strongly with PI, as well as with LL, albeit to a lower extent. This information should assist orthopaedic surgeons in the diagnosis and management of DLS.

## Additional file

Additional file 1: The dataset supporting the conclusions of this article. (XLSX 15 kb)

## Abbreviations

BMI: Body mass index
DLS: Degenerative lumbar spondylolisthesis
DS: Degenerative spondylolisthesis
LL: Lumbar lordosis
PI: Pelvic incidence
PT: Pelvic tilt
SS: Sacral slope
SVA: Sagittal vertical axis
TK: Thoracic kyphosis
